# Managing Non-Cancer Chronic Pain in Frail Older Adults: A Pilot Study Based on a Multidisciplinary Approach

**DOI:** 10.3390/ijerph20247150

**Published:** 2023-12-06

**Authors:** Teodora Figueiredo, Luís Midão, Rute Sampaio, Joana Carrilho, Constantino Coelho, Giovanni Cerullo, Antonella Di Paola, Angelo Carfì, Graziano Onder, Elísio Costa

**Affiliations:** 1 UCIBIO—Applied Molecular Biosciences Unit, Associate Laboratory i4HB-Institute for Health and Bioeconomy, Faculty of Pharmacy, University of Porto, 4050-313 Porto, Portugal; tgfigueiredo@ff.up.pt (T.F.); lmidao@ff.up.pt (L.M.); jcarrilho@ff.up.pt (J.C.); cccoelho@ff.up.pt (C.C.); 2 Porto4Ageing—Competences Centre on Active and Healthy Ageing, Faculty of Pharmacy, University of Porto, 4050-313 Porto, Portugal; 3 School of Medicine and Biomedical Sciences, University of Porto, 4050-313 Porto, Portugal; 4 Department of Biomedicine, Faculty of Medicine, University of Porto, 4200-319 Porto, Portugal; rutesampaio@med.up.pt; 5 CINTESIS@RISE, Faculty of Medicine, University of Porto, 4200-319 Porto, Portugal; 6 Palliative Care, Centro Hospitalar Universitário do Algarve, 8000-386 Algarve, Portugal; gcerullo@chalgarve.min-saude.pt; 7 Fondazione Policlinico Gemelli IRCCS, 00168 Rome, Italyangelo.carfi@policlinicogemelli.it (A.C.);; 8 Department of Geriatric and Orthopedic Sciences, Università Cattolica del Sacro Cuore, 00168 Rome, Italy

**Keywords:** chronic pain, frailty, older adults, intervention, case manager, health literacy, adherence, CHRODIS+

## Abstract

Considering the multidimensionality of chronic pain, it is crucial to develop comprehensive strategies for its effective management. However, establishing well-defined, evidence-based guidelines for such approaches remains challenging. To overcome this, we present the finding from a 4-month intervention to enhance the management of non-cancer chronic pain in older adults with pre-frailty and frailty. The intervention’s core elements comprised a multidisciplinary individualized plan, a case manager, and patient education. This pilot study involved 22 participants (≥65 years). It assessed changes in pain frequency and intensity (pain scale), frailty (Fried frailty phenotype criteria), and medication adherence (Brief Adherence Rating Scale) before and after the 4-month intervention. The results were encouraging: pain frequency and intensity and frailty score tended to decrease, and medication adherence showed significant improvement. This preliminary small-scale pilot study provides a foundation for further research and for exploring the potential scalability of this multidisciplinary patient-centred intervention.

## 1. Introduction

Chronic non-cancer pain refers to pain that endures for more than three months, is unrelated to any cancer-related condition [1], and exhibits a notable prevalence among the older population, estimated to range between 25% and 85% worldwide [2]. This increased burden among older adults has been partly attributed to the emergence and progression of chronic degenerative conditions associated with ageing, such as osteoarthritis. Chronic pain has the potential to diminish mobility, influence overall well-being and quality of life, contribute to cognitive decline, and even lead to accidents [3].

It is suggested that chronic pain can aggravate the challenges associated with ageing-related frailty in older individuals. Frailty, a geriatric syndrome, denotes a condition where an individual retains their independence but is exposed to a heightened risk of transitioning into a state of disability. Frailty shares a commonality with persistent pain in that both conditions become more prevalent as age advances [4]. Given the rapid population ageing, treatment needs to take cognisance of ageing-related conditions such as this one [5]. Moreover, patients with chronic pain are more likely to experience frailty than those who do not have chronic pain [6].

According to its definition, chronic pain cannot be eradicated entirely using conventional biomedical approaches. Instead, the focus shifts towards promoting effective coping strategies [7]. Nevertheless, there are barriers to geriatric pain management. For instance, non-adherence to treatment is frequent and often goes unnoticed by patients and their healthcare providers, ultimately leading to unfavourable clinical outcomes [8].

Nowadays, it is widely recognized that the management of chronic pain necessitates a comprehensive multidisciplinary approach. This approach encompasses pharmacological treatments and physical and psychological rehabilitation, as well as interventional techniques. These strategies are optimal for achieving effective pain control and improved outcomes, all while reducing the reliance on high-risk treatments such as opioids [9]. However, the absence of standardised guidelines and inconsistent approaches exacerbates the challenges of pain management. Individuals with chronic pain experience many biological, psychological, and social disturbances throughout their illness. Unfortunately, not all patients have access to specialised pain treatment units. Instead, they are commonly managed as if enduring an extended phase of acute pain, so various medications and interventions are tried to achieve a tolerable level of pain [7]. A recent study revealed a negative pain management index in 77% of chronic pain patients, signifying inadequate pain management in pain clinics and treatment centres [10].

Despite the growing evidence on non-cancer chronic pain management, it is still a prevalent problem that needs more attention and evaluation. There are several models and methodologies to create and implement interventions to address the challenges of chronic diseases, namely those outlined in the recent Integrated Multimorbidity Care Model proposed as part of the joint action (JA) on chronic diseases and promoting healthy ageing across the life cycle project—PLUS (CHRODIS+) project. CHRODIS+ stands for “implementing good practices for chronic diseases”. It is a European Union-funded project that aims to promote the exchange of knowledge and best practices in the field of chronic diseases. It is presently being embraced by 15 European pilot sites designing tailored programmes addressing non-communicable diseases in individuals [11,12]. Following the JA CHRODIS+ recommendations and criteria, the implementation strategy for an integrated care model begins with a scope analysis, primarily aimed at identifying the problem, defining the intervention’s purpose, and specifying the target population. Subsequently, a “strengths, weaknesses, opportunities, threats” (SWOT) analysis is conducted to pinpoint potential areas for improvement or intervention to incorporate into the integrated care model. Based on the identified improvement areas, a pilot action plan is developed, leading to the creation of an integrated care model tailored to address the specific challenge [11,12].

In this work, we aimed to develop an intervention based on the JA CHRODIS+ guidelines and run a small-scale preliminary pilot study to improve the management of non-cancer chronic pain in older adults with pre-frailty and frailty. The primary goal of this intervention was to reduce pain frequency and intensity by addressing healthcare fragmentation while improving patient education and treatment adherence. Moreover, a further aim was to test the feasibility of this approach and monitor patient engagement.

## 2. Materials and Methods

### 2.1. Study Design

A longitudinal pilot study was performed by the anaesthesiology service in the chronic pain consultation unit at Centro Hospitalar Universitário do Algarve (CHUA), Portugal. This study was authorised and approved ethically by the ethics committee of the CHUA (Nº UAIF 137/2022). All participants were duly asked to provide documented informed consent before their involvement. The rights and responsibilities of participants were comprehensively elucidated, and written consents were collected from all individuals.

Participants were recruited between 16th August 2022 and 5th September 2022. The sample comprised 22 older adults (≥65 years) diagnosed with non-cancer chronic pain, who agreed to participate in the study. Inclusion in the study required these participants to be considered pre-frail or frail, according to Fried’s frailty phenotype [13]. Older adults diagnosed with cancer or exhibiting moderate to severe cognitive deficits were excluded from the study as cognitive impairment can diminish the reporting of pain [14] and lead to inaccurate responses in self-perceived inquiries.

### 2.2. Development of the Intervention

To develop the intervention, the JA CHRODIS+ methodology was employed [11,12]. First, meetings were conducted with healthcare professionals from the pain consultation clinic to assess the service needs gaps and possibilities for improving the management of non-cancer chronic pain patients. Additionally, a national online survey was conducted among healthcare professionals, evaluating their needs and requirements. These steps were fundamental to the SWOT analysis, which led to our identifying the components described in Table 1.

Moreover, through the SWOT analysis, it was possible to identify potential improvement/intervention areas to include in the integrated care model. These areas were ranked based on priority, with the following order, from highest to lowest: technological solutions, education programmes, multidisciplinary teams, and case management. This strategic ranking process, combined with the insights gained from the SWOT analysis, formed the foundation for developing the integrated care model specifically designed to address the challenges of chronic pain in older populations.

### 2.3. Intervention

Following the creation of a personalized treatment plan for each participant by a multidisciplinary team of healthcare professionals at the study centre, each participant was assigned a case manager for the 4-month intervention. The case manager was responsible for coordinating, prioritising, and personalising the care plan. The case manager established a close and attentive rapport with the patient, fostering an atmosphere of proximity. The patient had a direct line number, allowing them to always be able to contact the pain consultation service. At least 2 follow-up calls were conducted by the case manager, accompanied by an intermediate consultation to make necessary adjustments to treatment and medication plans. Moreover, the case manager played a role in informing and educating the patient about their health condition and the importance of treatment adherence, a balanced diet, and physical exercise to facilitate informed decision-making. This encompassed decisions regarding pain prevention or management options, which could also improve treatment adherence.

The case managers were nurses. A total of three nurses were randomly distributed among the participants.

### 2.4. Outcome Assessment

During the 4-month intervention, the case manager conducted two evaluation moments for each participant: prior to implementation (pre-intervention) and upon completion (post-intervention). The pain, frailty, and medication adherence levels were measured at each of these moments. Demographic data (age, gender, marital status, education level, and socioeconomic level) were acquired at the recruitment phase. Regarding gender, participants were asked to identify themselves as male, female, or “other”. Concerning marital status, options included single, married, divorced, or widowed. As for education, participants were queried about their educational background, whether they had incomplete elementary education, completed elementary education, middle school education, high school education, or college degree. To acquire information about socioeconomic status, participants were asked the following question: do you think you have enough money to meet your needs?

Pain frequency and intensity were assessed using the pain scale [14]. Participants rated the frequency of their pain as either “1—no pain”, “2—pain present, but not manifested in the last 3 days”, “3—pain present 1 to 2 days in the last 3 days”, or “4—pain present every day for the last 3 days”. Regarding pain intensity, the participants ranked “1—no pain”, “2—medium pain”, “3—moderate pain”, “4—severe pain”, or “5—the pain is unbearable at times”.

The level of frailty was assessed based on the Fried frailty phenotype criteria [13]. Adjusting these criteria, a phenotype of pre-frailty was identified by the fulfilment of at least one of the following components (score 1–2) and a phenotype of frailty by the presence of at least 3 components (score ≥ 3):

- Shrinking: unintentional weight loss equal to or exceeding 5% of body weight during the preceding year. The patient was queried about whether they experienced a loss of 6 kg or over in the past 6 months or 3 kg or over in the past month without deliberate dietary or exercise measures. An affirmative response was assigned a score of 1, while a negative response received a score of 0.
- Weakness: determined by grip strength in the lowest 20% at baseline, with adjustments made for gender and body mass index quartiles. If the grip strength exceeded this threshold, a score of 0 was allocated; otherwise, a score of 1 was assigned.
- Poor endurance and energy: indicated by self-reported feelings of exhaustion. The patient was asked whether they experienced a sensation of lack of energy. If they answered affirmatively, a score of 1 was assigned; if they answered negatively, a score of 0 was given.
- Slowness: the slowest 20% of the population was defined at baseline, based on time to walk 6 m, adjusting for gender and height. If the participant could complete the task in less than 10 s, a score of 0 was assigned; if not, a score of 1 was given.
- Low physical activity level: ascertained through patient inquiry regarding regular engagement in physical activities that require a low or moderate level of energy, such as gardening, household or car cleaning, or going for a walk. If the response was “once or twice a week”, it corresponded to a score of 0, while answering “a few times a month or hardly ever” resulted in a score of 1.

Medication adherence was determined using the Brief Adherence Rating Scale (BARS) [8]. Comprising 4 items, the BARS encompasses 3 questions and an overarching visual analogue rating scale. This scale evaluates the portion of doses consumed by the patient in the study period, ranging from 0% to 100%. The three questions investigate the patient’s awareness of their medication regimen and episodes of missed dosages. Specifically, the questions address the number of prescribed doses per day, the number of days in the study period when the patient did not take the prescribed doses, and the number of days the patient took fewer doses than prescribed.

### 2.5. Data Analysis

Gender, marital status, education level, socioeconomic level, and patient adherence behaviour were reported as frequencies (% (number of patients)). Age (as the only variable with normal distribution) was presented as mean ± SD, while frailty status, medicine adherence, pain frequency, and pain intensity were reported as the median and interquartile range (median (IQR)) (due to their non-normal distribution).

The BARS scale was used to assess medication adherence. We assessed the degree of adherence for each medicine that each patient took—medicine adherence—and the degree of patient medicine adherence behaviour—patient adherence behaviour. For values lower than 80%, the patient adherence behaviour was classified as non-adherent, while for values equal or more than 80%, the patient adherence behaviour was classified as adherent [15].

Kolmogorov–Smirnov statistics were used to evaluate sample normality distribution.

To compare pre- and post-intervention, we used the Wilcoxon test for the frailty status and medicine adherence (as these continuous variables did not follow a normal distribution). For the categorical ordinal variables, such as pain frequency and intensity, we also used the Wilcoxon test. For comparison between groups for the patient adherence behaviour, the only categorical nominal variable, we used the McNemar test. *p* values < 0.05 were considered significant. Descriptive analyses were performed using SPSS Statistics software, version 28.0.

## 3. Results

### 3.1. Sample Description

The sample comprised 22 participants, with males accounting for 18.2%. The participants’ ages ranged from 66 to 86 years, with an average age of 74.68 ± 6.21 years. Most participants were married (63.6%) and had either incomplete or completed elementary school education (59.0%). Most of them indicated having a low (50.0%) or moderate (45.5%) socioeconomic status (Table 2).

### 3.2. Frailty Assessment

Following the intervention, the average frailty score (*n* = 22) slightly decreased, from 4 (3–4.25) to 4 (3–4) (Table 3). Of the 22 participants, 9 experienced a reduction in their frailty score, 8 remained unchanged, and 5 showed an increase (Table 4).

### 3.3. Pain Assessment

Concerning pain evaluation, minor discrepancies were observed in the ratings of pain frequency and intensity (Table 3). Post-intervention, among the 22 participants, 3 reported reduced pain frequency, 17 noted no change, and 2 indicated increased pain frequency. Regarding pain intensity, 10 reported lower intensity, 6 remained unchanged, and 6 reported heightened pain intensity (Table 4).

### 3.4. Medicine Adherence and Patient Adherence Behaviour Assessment

Medicine adherence was assessed both prior to and after the intervention. However, due to changes in medication regimens during the intervention for two patients, a comparison and tracking of their adherence between these two moments was not possible.

Considering only the medications that remained consistent throughout the intervention, the remaining participants (*n* = 20) exhibited an average medication count of 4.65 ± 3.23, ranging from a minimum of 1 to a maximum of 12. Using 80% as the cut-off point, prior to the intervention, 35% of participants were non-adherent, a value that decreased drastically after the intervention to 5% of non-adherent participants (Table 3), making this change statistically significant (*p* = 0.031).

Out of the 93 medications examined, the median adherence rate before the intervention was 100 (80–100), while after the intervention it improved to 100 (100–100), also a statistically significant change (*p* = 0.011). Furthermore, the category of medications that demonstrated the most enhancement in adherence rates was the group targeting the nervous system.

## 4. Discussion

Aiming to enhance the management of non-cancer chronic pain in older adults, an intervention was developed based on the guidelines and criteria outlined in the Integrated Multimorbidity Care Model proposed by the JA CHRODIS+ project [11,12]. This is the first time this methodology has been used to address chronic pain in older populations.

Through a comprehensive analysis, it was possible to identify several issues with the current landscape of chronic pain management in Portugal. These included fragmentation of care, particularly among the older population, poor coordination and organisation, and elevated frustration levels among patients and healthcare professionals. In response to these findings, an integrated care model was created whose core elements comprised a multidisciplinary, individualised treatment plan, case management, and patient education.

This paper reports the results of a pilot study involving 22 frail older adults, each of whom received an individualised treatment plan and was paired with a case manager for a 4-month duration. The role of the case manager extended beyond mere coordination and education; they actively engaged with the participants to address their healthcare needs ensuring a holistic approach to care, while also monitoring progress and refining treatments.

Out of the 22 patients in the sample, only four were male. This gender disparity can be partly attributed to women’s higher susceptibility to multiple chronic pain disorders than men [16]. Additionally, women exhibit distinct pain sensitivity and perception patterns [16].

After the 4-month intervention, evaluations were made to determine pain, frailty, medicine adherence, and patient adherence behaviour changes. Notably, there was a tendency for both pain frequency and intensity to decrease (Table 3). The frailty score measurement was justified by the well-established association between chronic pain and the exacerbation of frailty in older adults [17]. Consequently, as pain levels tended to diminish with the intervention, frailty also exhibited improvement. In fact, among the 22 participants, 17 either decreased or maintained their frailty score (Table 4). This outcome held significance as frailty tends to escalate quickly with age [18]. Additionally, since frailty entails higher costs related to the utilisation of healthcare resources, this intervention holds economic advantages. Transitioning from frail to a pre-frail condition alone reduces the average cost of healthcare resources per year [19].

Medication adherence is negatively influenced by complex regimens, a need to understand the illness and its complications, and physical and financial challenges [20]. This intervention aimed to enhance medicine adherence and patient adherence behaviour by educating patients about their condition and treatment. Encouragingly, there was a significant increase in medication adherence (Table 3). Moreover, the category of medications that showed the most enhancement in adherence rates was the group targeting the nervous system. This can be explained because the classes of drugs most commonly used in treating non-cancer chronic pain, such as opioids, target the central nervous system [21]. The observed increase aligns with previous findings, which showed that medication adherence tends to rise when individuals experience symptom relief [15].

While the study showed promising results, it has some limitations. It is important to note that most of these improvements did not reach statistical significance in our analysis (*p* > 0.05). This research was also conducted as a pilot study on a smaller scale with 22 participants, potentially limiting its representation of the broader population. Moreover, the intervention’s duration was four months, which did not allow for the observation of long-term effects. The study also had a significant gender imbalance, which may impact the generalizability of the results as chronic pain experiences and responses can differ between genders. Finally, the study did not compare the intervention to existing treatments or control groups, making it challenging to determine the relative effectiveness compared to other existing approaches.

Given the established multifaceted nature of chronic pain, several treatment programmes have been developed and tested, extending beyond medication alone. While some of these programmes have shown improvements in pain intensity, the availability of clear evidence-based guidelines still needs to be improved. Further research is required to pinpoint the components and combinations that would provide optimal benefits for individuals with chronic pain [22].

We anticipate that this study will offer an enhanced guideline for managing non-cancer chronic pain in older adults. Additionally, this report shows and proves the feasibility of an approach to pain based on the Integrated Multimorbidity Care Model proposed by the JA CHRODIS+ project. The methods outlined in this project involve several key steps to achieve its objectives, which include promoting integrated care for chronic diseases, sharing knowledge and best practices, and advocating for policy recommendations. In this communication, we report the results after the pilot programme’s implementation, where we tested and evaluated the effectiveness of the intervention, which showed promising results. We strongly believe that this success paves the way for the next step, which is the scaling up of the intervention.

In conclusion, the multidisciplinary, patient-centred approach outlined demonstrates a noticeable trend toward reducing frailty and alleviating pain intensity and frequency. Moreover, there was a significant increase in medicine adherence and patient adherence behaviour after the 4-month period. This approach acknowledges the complexity of chronic pain and recognises that addressing various dimensions of treatment can lead to improved outcomes. This preliminary small-scale pilot study has revealed encouraging and optimistic findings, suggesting that the methodology and intervention under investigation hold great potential. As a result, there is a strong rationale for considering expanding the utilisation of this approach in a more extensive and rigorous clinical trial in the future.

## Figures and Tables

**Table 1:** Components identified in the SWOT analysis.

**Strengths**	**Weaknesses**
- Teams are multidisciplinary, aiming for a holistic patient intervention, including social and psychological dimensions. - Professionals are highly qualified to work in a pain context and have a scientific background and expertise in chronic pain management.	- Poor care coordination and organization. - Lack of integrated evaluation of older patients, including with community, and fragmentation of care - Perceived difficulties in monitoring, validation, implementation, and management of interventions. - Frustration from patients and healthcare professionals. - Unnecessary referrals. - Lack of functional rehabilitation
**Opportunities**	**Threats**
- Expertise and highly skilled professionals. - Extend therapeutical plans to include non-pharmacological therapies, complementary therapeutics, and functional rehabilitation. - Improve communication skills and medication adherence through improvement of health literacy.	- Frustration and exhaustion of professionals and patients; - Health system factor dimensions; - Lack of support to caregivers.

**Table 2:** Demographic characteristics of the sample (*n* = 22).

Demographic Characteristics	Sample (*n* = 22)
Age, years ± SD	74.68 ± 6.21
Gender, % (*n*)	
Male	18.2 (4)
Female	81.8 (18)
Marital status, % (*n*)	
Single	4.5 (1)
Married	63.6 (14)
Divorced	9.1 (2)
Widower	22.7 (5)
Education level, % (*n*)	
Incomplete or completed elementary school	59.0 (13)
Middle school	27.3 (6)
High school	9.2 (2)
College degree	5.5 (1)
Socioeconomic level, % (*n*)	
Low	50.0 (11)
Moderate	45.5 (10)
High	4.5 (1)

**Table 3:** Assessment of frailty, frequency and intensity of pain, medicine adherence, and patient adherence behaviour: comparison between pre- and post-intervention.

Patient’s Characteristics	Pre-Intervention	Post-Intervention	*p*-Value
Frailty, median (IQR)	4 (3–4.25)	4 (3–4)	0.512
Pain frequency, median (IQR)	4 (4–4)	4 (3.75–4)	0.480
Pain intensity, median (IQR)	3 (3–4)	3 (2–4)	0.422
Medicine Adherence, median (IQR)	100 (80–100)	100 (100–100)	0.011
Patient adherence behaviour, % (*n* of patients)			0.031
adherent	65 (13)	95 (19)	
non-adherent	35 (7)	5 (1)	

**Table 4:** Categorized changes in frailty and pain frequency and intensity after the intervention among the participants.

Outcome Changes	Frailty, % (*n*)	Pain Frequency, % (*n*)	Pain Intensity, % (*n*)
worsening	22.7 (5)	9.1 (2)	27.3 (6)
no difference	36.4 (8)	77.3 (17)	27.3 (6)
improvement	40.9 (9)	13.6 (3)	45.5 (10)

## Data Availability

The data presented in this study are available on request from the corresponding author. The data are not publicly available due to privacy reasons.
